# Effects of the duration of hyperlipidemia on cerebral lipids, vessels and neurons in rats

**DOI:** 10.1186/s12944-016-0401-6

**Published:** 2017-01-31

**Authors:** Weichun Yang, He Shi, Jianfen Zhang, Ziyi Shen, Guangyu Zhou, Minyu Hu

**Affiliations:** Department of Nutrition and Food Hygiene, School of Public Health, Central South University, Changsha, 410078 China

**Keywords:** Duration time, Hyperlipidemia, Cerebral lipid, Cerebral vessels, Neurons

## Abstract

**Background:**

The present study was designed to investigate the effects of hyperlipidemia on the cerebral lipids, vessels and neurons of rats, and to provide experimental evidence for subsequent intervention.

**Method:**

One hundred adult SD rats, half of which were male and half of which were female, were randomly divided into five groups on the basis of serum total cholesterol (TC) levels. Four groups were fed a hypercholesterolemic diet (rat chow supplemented with 4% cholesterol, 1% cholic acid and 0.5% thiouracil – this is also called a CCT diet) for periods of 1 week, 2 weeks, 3 weeks and 4 weeks, respectively. A control group was included. The levels of serum lipids, cerebral lipids, free fatty acids (FFA), interleukin-6 (IL-6), interleukin-1 (IL-1), tumor necrosis factor alpha (TNF-α), vascular endothelial growth factor (VEGF), oxidized low density lipoprotein (ox-LDL), A-beta precursor proteins (APP), amyloid beta (Aβ), glial fibrillary acidic protein (GFAP) and tight junction protein Claudin-5 were measured after the experiment. The pathologic changes and apoptosis of the rat brains were evaluated.

**Results:**

Compared with the control group, after 1 week of a CCT diet, the levels of serum total cholesterol (TC), triglycerides (TG), low density lipoprotein cholesterol (LDL-C) and brain triglycerides had increased by 2.40, 1.29 and 1.75 and 0.3 times, respectively. The serum high density lipoprotein cholesterol (HDL-C) had decreased by 0.74 times (*P* < 0.05) and the expression of IL-1, TNF-α and GFAP in the brains had increased (*P* < 0.05). In the second week, the expression of FFA and APP in the brains, and the amount of apoptotic neurons, had increased (*P* < 0.05). In the third week, the levels of VEGF, Ox-LDL and Aβ had increased, and the expression of Claudin-5 had decreased in the brains (*P* < 0.05). In the fourth week, the levels of TC, LDL-C and the amount of apoptotic neurons had increased (*P* < 0.05). The correlation analysis showed a positive correlation among FFA, TNF-α, VEGF, ox-LDL, Aβ, GFAP and neuronal apoptosis in the rat brains, and they all were negatively correlated with Claudin-5 (*P* < 0.05).

**Conclusion:**

Hyperlipidemia may activate astrocytes by means of high levels of TG that will have direct toxic effects on the cerebral vessels and neurons by causing the secretion of TNF-α and IL-1 in the brains of rats. In the metabolic procession, brain tissue was shown to generate FFA that aggravated the biosynthesis of ox-LDL. With the extension of the duration of hyperlipidemia, high levels of cerebral TC and LDL-C were shown to aggravate the deposition of Aβ, induce the secretion of VEGF, reduce the expression of tight junction protein Claudin-5 and change the permeability of blood–brain barriers to factors that could damage cerebral vessels and neurons.

## Background

Hyperlipidemia is a kind of lipid metabolism disorder characterized by elevated serum total cholesterol (TC), low-density lipoprotein cholesterol (LDL-C), triglyceride (TG) levels and/or decreased high-density lipoprotein cholesterol (HDL-C). There has been an increase in awareness of this condition among scientists. It is now recognized that hyperlipidemia can be severely detrimental as a major risk factor for atherosclerosis, cardio-cerebrovascular disease, fatty liver and stroke [1]. It is also related to neurodegenerative diseases such as Alzheimer’s Disease and Niemann-Pick disease [2–4]. Human epidemiological studies have revealed that high cholesterol intake increases the risk of impaired cognitive function [5, 6], and neuroimaging has shown inverse associations between triglyceride levels and cerebral blood flow [7]. The significant negative effects of a high-fat/high-cholesterol diet on hippocampal morphology have also been demonstrated in animal research, including elevated microglial activation and reduced neuronal development in [8]. Because of the complexity of the structure and function of the central nervous system, the present study was designed to elucidate the effects of hyperlipidemia on the cerebral lipids, vessels and neurons in rats with hyperlipidemia. Analyzing the results may provide laboratory evidence for subsequent anti-injury research.

## Methods

### Composition of diets

The standard diet given to the rats consisted of the following ingredients: 20% wheat, 20% rice, 10% corn, 24% soybean cake, 10% fish flour, 10% wheat bran, 1% salt, 3% bone meal and 1% multivitamins; the diet was purchased ready-made from the Department of Zoology, Xiangya School of Medicine, Central South University. The hypercholesterolemic diet consisted of 94.5% of the standard diet, plus 4% cholesterol, 1% cholic acid and 0.5% thiouracil [9].

### Experimental animals and design

One hundred healthy adult Sprague–Dawley rats with a body weight of 227.33 ± 11.68 g, half male and half female, were purchased from the Department of Zoology, Xiangya School of Medicine, Central South University. After 1 week of adaptation to the standard diet, the rats were divided into five groups with similar distributions of serum cholesterol levels (*n*=20 per group) based on the obtained level of TC. Group I served as the control group and was fed a standard diet. Groups II to V comprised rats that were fed the hypercholesterolemic (CCT) diet for periods of 1 week, 2 weeks, 3 weeks and 4 weeks, respectively. The rats were raised in separate cages with daylight illumination and given water ad libitum. The food intake was recorded everyday, and it was restricted to 20 ± 2 g per day in order to prevent excessive weight gain. The temperature in the animal room was controlled at 24 ± 2 °C, and the humidity was kept at 65–70%.

The rats were euthanized with an i.p. injection of sodium pentobarbital (45 mg · kg · bw^−1^); all blood samples were obtained from the abdominal aorta. Next, the brains were removed and divided sagittally, the right hemisphere was used for morphology, and the left hemisphere was used for biochemical measurements. Cortex samples were collected without contamination. They were weighed and homogenized in a basic solvent that was determined to be appropriate according to the biochemical indicators (10 volumes of phosphate-buffered saline for enzyme-linked immunosorbent assay or methanol for lipid detection), and then they were centrifuged at 100,000 *g* for an hour to collect the membranes. All animal experimental procedures were performed in accordance with the guidelines of the animal ethical committee for animal experimentation in China.

### Detection methods and quality control

The levels of TC and TG in the serum and in the brain were measured using enzymatic methods of cholesterol oxidase-peroxidase-4-aminoantipyrine (COD-PAP) and glycerol phosphate oxidase-peroxidase-4-aminoantipyrine (GPO-PAP), respectively. The levels of HDL-C were determined after the precipitation of lipoprotein-B using phosphototungstic acid/Mg^2+^ (PTA/Mg^2+^), and the concentration of LDL-C was calculated. The content of free fatty acid (FFA) in the brain was detected using the colorimetric method with a copper reagent. The concentrations of interleukin-1 (IL-1), interleukin-6 (IL-6), tumor necrosis factor alpha (TNF-α), vascular endothelial growth factor (VEGF), oxidized low density lipoprotein (ox-LDL) and amyloid beta (Aβ) in the brain were all determined using a biotin-avidin–based enzyme-linked immunosorbent assay kit (ELISA). Western blot analysis was used to determine the levels of A-beta precursor proteins (APP), glial fibrillary acidic protein (GFAP) and tight junction protein Claudin-5 in the brains. The morphologic changes in the hippocampal neurons and apoptosis were evaluated using the method of Nissl staining and Terminal deoxynucleotidyl transferase-mediated dUTP-biotin nick end labeling assay (TUNEL). Each form of apparatus used in the experiment was washed and disinfected. The instruments were debugged and calibrated before being used, and a pilot practice with distilled water was performed before the experiment itself was conducted. All testing was implemented in accordance with the the instructions of the experiments, and the experimental data was double-recorded, input and checked.

### Statistical analysis

All analysis was carried out with SPSS 18.0 software. Quantitative data was expressed as a mean ± standard deviation. Two-way ANOVA factorial analysis was used to measure diet and duration. Normality tests and homogeneity of variance tests were performed. Differences among groups were analyzed using the ANOVA or Kruskal-Wallos *H* tests, followed by post hoc Student-Newman-Keuls (SNK) tests or Bonferroni tests. Pearson correlation or Spearman rank correlation analysis was used for correlation analysis. Probability values of less than 5% (*P* < 0.05) were considered significant.

## Results

During the experiment, the rats performed well in terms of water drinking, diet consumption and defecation, and their body weight changes and activities were normal.

### Serum lipid parameters

Factorial analysis showed there was significant difference in the levels of plasma TC (*F* = 23.181, *P* = 0.000), TG (*F* = 15.384, *P* = 0.000), LDL-C (*F* = 509.581, *P* = 0.000) and HDL-C (*F* = 26.538, *P* = 0.000) at different stages of the experiment. The plasma lipid test results are shown in Table 1.

**Table 1:** Plasma lipids in different groups (*n*=20, $\overline{x}\pm \mathrm{S}\mathrm{D}$, mmol/L)

Indictors	Group I Control	Group II CCT diet for 1 week	Group III CCT diet for 2 weeks	Group IV CCT diet for 3 weeks	Group V CCT diet for 4 weeks	F/H	*P-*Value
TC	1.90 ± 0.19^a^	6.47 ± 1.58^b^	11.15 ± 4.01^c^	15.69 ± 2.31^d^	19.55 ± 1.66^e^	86.581	0.000
TG	0.48 ± 0.12^a^	1.10 ± 0.42^b^	1.54 ± 0.47^c^	2.16 ± 0.37^d^	3.21 ± 0.62^e^	83.580	0.000
LDL-C	0.55 ± 0.06^a^	1.51 ± 0.04^b^	2.06 ± 0.08^c^	2.85 ± 0.05^d^	3.04 ± 0.07^e^	94.963	0.000
HDL-C	1.00 ± 0.21^a^	0.74 ± 0.03^b^	0.68 ± 0.04^b^	0.42 ± 0.04^c^	0.36 ± 0.05^c^	131.665	0.000

*Note:* statistics with underlined data indicate the *H* value; otherwise, the figure indicates the *F* value. Different superscript letters in each row indicate significant differences among groups (*P* < 0.05)

Table 1 is a comparison against the control group, and it shows that the levels of serum TC, TG and LDL-C dramatically increased by 2.40, 1.29 and 1.75 times, respectively, in group II (*P* < 0.05), and the levels of serum HDL-C decreased by 0.26 times (*P* < 0.05). Post hoc tests showed significant differences in the levels of serum TC, TG, LDL-C and HDL-C at different times in rats fed the CCT diet, and it was found in particular that serum TC, TG and LDL-C levels increased and serum HDL-C levels decreased with the extension of feeding time.

### Cerebral lipid parameters

Factorial analysis shows a significant difference in the levels of cerebral TC (*F* = 4.013, *P* = 0.010), LDL-C (*F* = 4.993, *P* = 0.003) and HDL-C (*F* = 8.196, *P* = 0.000) at different stages of the experiment. The cerebral lipid results are shown in Table 2.

**Table 2:** Cerebral lipid in different groups (*n*=20, $\overline{x}\pm \mathrm{S}\mathrm{D}$, mg/g)

Indictors	Group I Control	Group II CCT diet for 1 week	Group III CCT diet for 2 weeks	Group IV CCT diet for 3 weeks	Group V CCT diet for 4 weeks	F/H	*P-*Value
TC	17.34 ± 1.43^a^	17.52 ± 1.45^a^	17.85 ± 1.34^a^	18.24 ± 1.24^a^	19.54 ± 1.12^b^	8.749	0.000
TG	1.51 ± 0.13^a^	1.96 ± 0.23^b^	2.20 ± 0.48^b^	2.49 ± 0.84^b^	2.41 ± 0.87^b^	35.737	0.000
LDL-C	3.49 ± 1.10^a^	4.30 ± 1.18^a^	4.58 ± 1.61 ^a^	4.71 ± 1.31^a^	8.24 ± 2.43^b^	26.158	0.000
HDL-C	31.14 ± 5.81^a^	30.08 ± 2.85^a^	20.24 ± 3.06^b^	18.26 ± 2.02^b^	15.39 ± 1.80^c^	73.666	0.000

*Note:* statistics with underlined data are for the *H* value; otherwise, they are for the *F* value. Different superscript letters in each row indicate significant differences among groups (*P* < 0.05)

Table 2 indicates that compared with the control group, the levels of brain TG increased by 0.3 times in group II (*P* < 0.05). There was no significant change in the level of brain TC, LDL-C or HDL-C in the group fed the CCT diet for 1 week (*P* < 0.05). A two-group comparison shows that the levels of brain TC and LDL-C in group V were higher than in groups II to IV (*P* < 0.05). However, there was no significant difference in the mean concentration of brain TC or LDL-C in groups II, III and IV (*P* < 0.05). Also, the levels of brain HDL-C in groups III to V were lower than in group II; group V was the lowest (*P* < 0.05).

### Cerebral biochemical examination

Factorial analysis shows there was a significant difference in the levels of cerebral FFA (*F* = 6.358, *P* = 0.001), IL-1 (*F* = 7.790, *P* = 0.000), Aβ (*F* = 40124, *P* = 0.009), VEGF (*F* = 8.270, *P* = 0.000) and ox-LDL (*F* = 8.730, *P* = 0.000) at different stages of the experiment. The cerebral biochemical examination results in different groups are shown in Table 3.

**Table 3:** Cerebral biochemical examination in different groups (*n*=20, $\overline{x}\pm \mathrm{S}\mathrm{D}$)

Indicators	Group I control	Group II CCT diet for 1 week	Group III CCT diet for 2 weeks	Group IV CCT diet for 3 weeks	Group V CCT diet for 4 weeks	F/H	*P-*Value
FFA (μmol/g)	472.65 ± 193.38^a^	579.90 ± 95.18^a^	770.41 ± 144.48^b^	1037.99 ± 377.16^b^	1438.90 ± 326.22^c^	65.801	0.000
IL-6 (ng/L)	147.51 ± 28.34^a^	156.86 ± 20.48^a^	178.49 ± 29.25^b^	192.56 ± 24.16^bc^	198.94 ± 21.44^c^	15.850	0.000
IL-1 (μg/L)	161.57 ± 21.10^a^	193.81 ± 38.81^b^	214.73 ± 17.53^b^	220.41 ± 23.30^b^	246.32 ± 18.84^c^	54.777	0.000
TNF-α (ng/L)	196.82 ± 21.40^a^	213.26 ± 32.64^b^	222.68 ± 22.00^b^	245.74 ± 31.04^b^	220.01 ± 21.19^c^	9.151	0.000
Aβ (μg/L)	1639.70 ± 173.51^a^	1714.77 ± 261.51^a^	1666.79 ± 235.28^a^	2140.96 ± 266.53^b^	2182.38 ± 201.22^b^	22.431	0.000
VEGF (pg/L)	1142.21 ± 135.13^a^	1261.86 ± 216.36^a^	1465.40 ± 316.07^a^	1839.56 ± 231.65^b^	2067.00 ± 346.23^b^	63.595	0.000
Ox-LDL (μg/L)	45.36 ± 4.66^a^	47.45 ± 9.00^a^	50.59 ± 6.51^a^	70.34 ± 11.07^b^	65.91 ± 6.04^b^	47.675	0.000

*Note:* statistics with underlined data represent the *H* value; otherwise, they indicate the *F* value. One indicator in the same row with different superscript letters indicates significant differences among groups (*P* < 0.05), and the outcome with the same superscript letters suggests that the differences among the groups was not statistically significant

Table 3 shows that the levels of cerebral IL-1 and TNF-α increased significantly in group II compared with the control group (*P* < 0.05). There was no significant difference in the levels of brain FFA, IL-6, VEGF in II group (*P* < 0.05). A two-group comparison shows that the levels of brain FFA in groups III to V were higher than in group II, with group V being the highest (*P* < 0.05). The levels of cerebral IL-1 in group V were higher than in groups II to IV (*P* < 0.05), yet there was no significant difference among groups II to IV (*P* > 0.05). Also, the levels of cerebral TNF-α, Aβ, VEGF and ox-LDL in groups IV and V were all higher than in groups II and III (*P* < 0.05).

### Cerebral GFAP, APP and Claudin-5

**Fig. 1:**
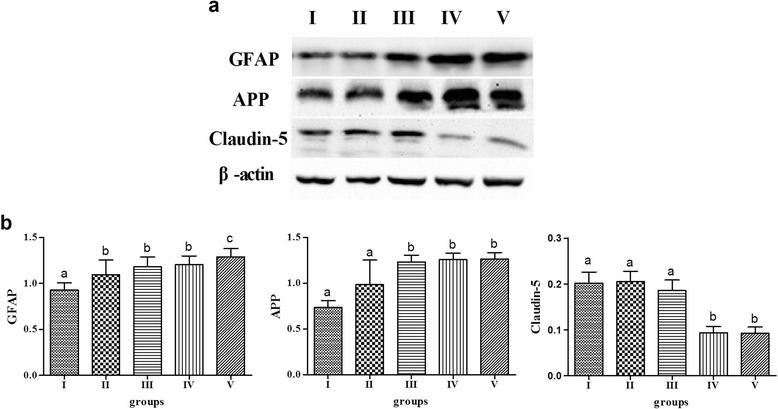
Effects of hyperlipidemia on cerebral GFAP, APP and Claudin-5 in rats. **a** The protein expressions of cerebral GFAP, APP and Claudin-5 in respective groups. **b** The expression ratio of cerebral GFAP, APP and Claudin-5 to actin was determined based on quantification of the relative density of western blotting bands, *n*=20, values are expressed as the mean ± SD, and bars without a common superscript letter differ significantly (*P* < 0.05)

The levels of cerebral GFAP, APP and Claudin-5 in all the groups were measured using western blot analysis [10, 11] (Fig. 1). The expression of the cerebral protein GFAP was higher in group II compared with the control group (*P* < 0.05), but it was not higher in the expression of protein APP or Claudin-5 (*P* > 0.05). The two-group comparison indicated that the relative intensity of GFAP in group V was more intense than in groups II to IV, but there was not a significant difference among groups II to IV (*P* > 0.05). The expression of cerebral APP in groups III to V was greater compared with that of group II (*P* < 0.05), with no significant difference among groups III to V (*P* > 0.05). The mean intensity of Claudin-5 in groups IV and V was more intense than that found in groups II and III (*P* < 0.05).

### Morphologic changes of hippocampus in rats

**Fig. 2:**
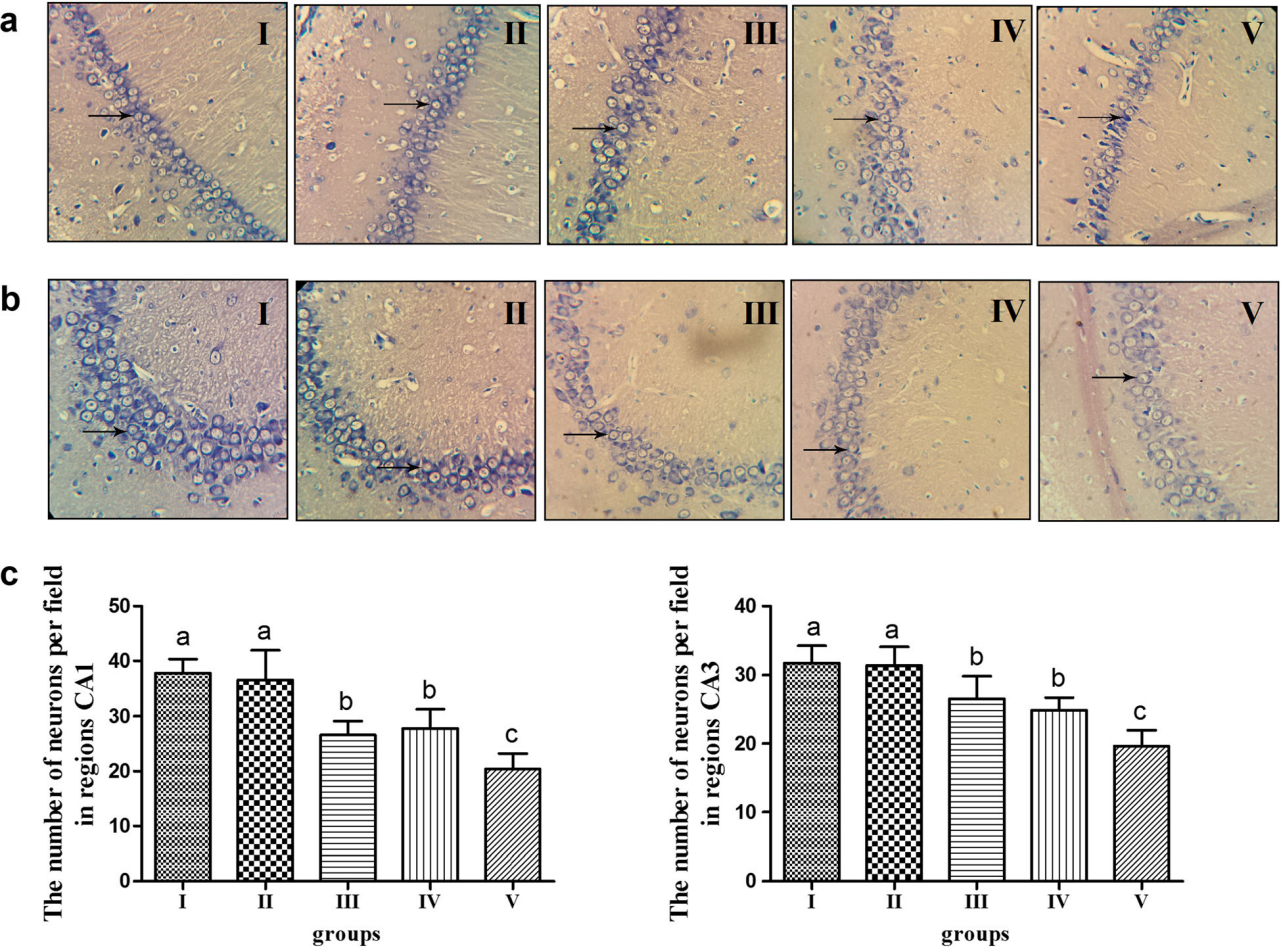
Morphologic changes of hippocampus stained by Nissl staining using a light microscope (magnification 400×) and counting the results. **a** Nissl staining of region CA1 of hippocampus in different groups. **b** Nissl staining of region CA3 of hippocampus in different groups. **c** The number of neurons per field in regions CA1 and CA3 of hippocampus. *n*=20, values are expressed as the mean ± SD. Bars without a common superscript letter differ significantly (*P* < 0.05)

Nissl staining was used to detect the morphologic changes of hippocampus in rats and the count of neurons in regions CA1 and CA3 were measured (five sections from each group were selected randomly and counted using Image-ProPlus software) [12] (Fig. 2). The normal morphological neuron-cells in the control group were arranged densely with blue Nissl granules. In the rats of group II, the structures of the pyramidal cells were regularly arranged and the hippocampal neurons had many visible blue-strained Nissl granules. The changes were slight in group III, with neurons loosely arranged and bulging, and the Nissl granules were reduced. There were typical neuron lesions in group IV, which we believe resulted from hippocampal neuron loss, and they were loosely arranged and slightly shrunk. The hippocampal neurons were evidently wrinkled in group V, and the phenomenon of cytolysis was distinctly evident. Given the resulting count, the number of neurons in regions CA1 and CA3 of the hippocampus in group II had no significant difference as compared with the control group. Post hoc tests indicated the amount of neurons in regions CA1 and CA3 in groups III to V were lower than those in group II; group V was the lowest (*P* < 0.05).

### Assay of TUNEL in hippocampus

**Fig. 3:**
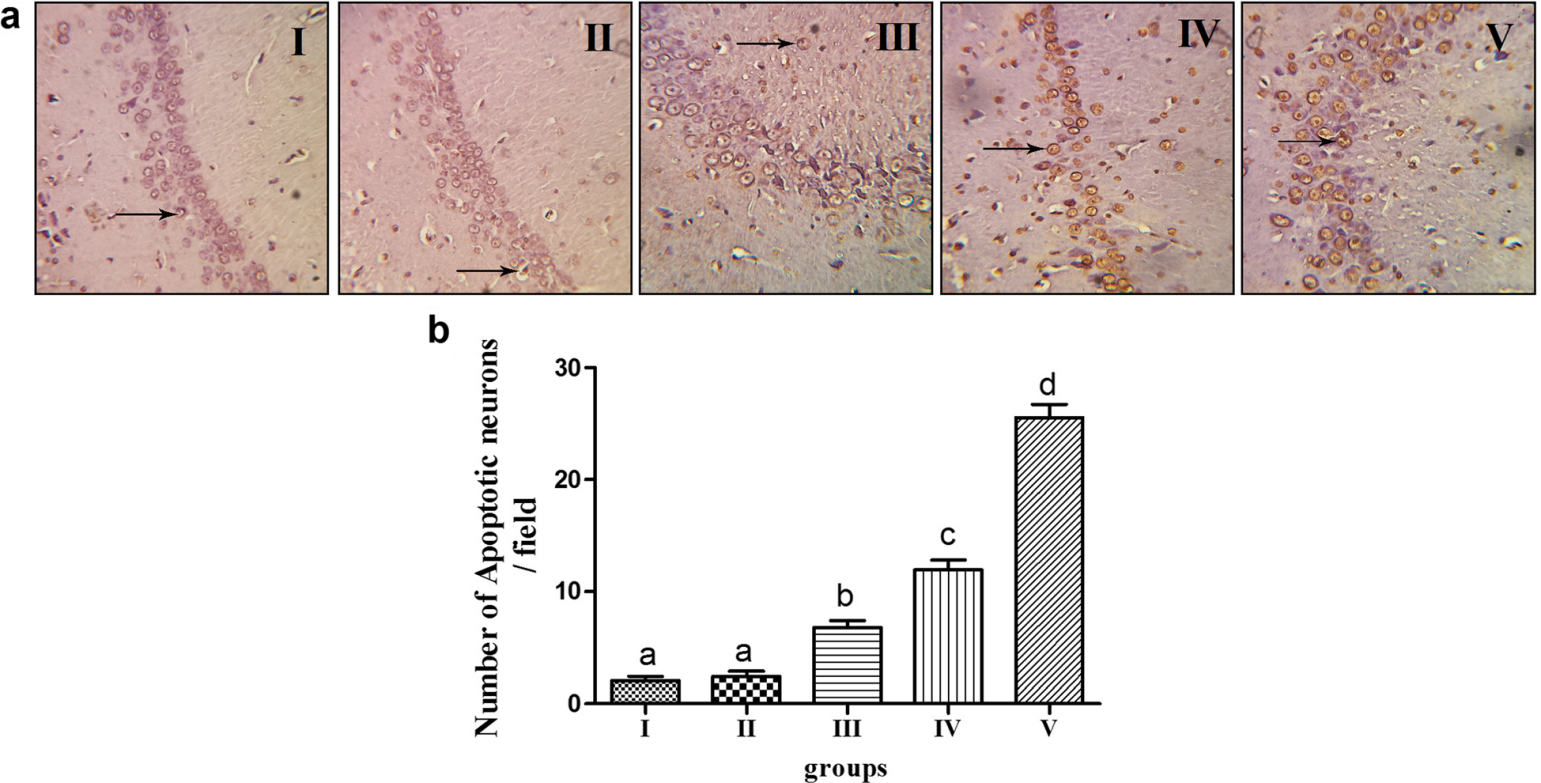
Morphologic changes in the hippocampus detected by TUNEL using a light microscope (magnification 400×) and the resulting counts. **a** TUNEL assay of region of hippocampus in different groups. **b** The number of apoptotic neurons per fields in hippocampus. *n*=20, values are expressed as the mean ± SD. Bars without a common superscript letter differ significantly (*P* < 0.05)

An assay of the TUNEL test was used to evaluate the damage of hyperlilpidemia on neurons and the count of apoptotic neurons was measured (selecting five sections from each group randomly and choosing 3 fields of view to calculate the number of positive cells, in order to calculate the average number of apoptotic cells per unit area). Through light microscope observation, the TUNEL assay results in Fig. 3 showed that the positive apoptotic cells were brown-yellow. The photomicrographs of the control group revealed tightly arranged neurons in the hippocampus of the rats and few positive apoptotic cells of the brown-yellow color. In group II, the hippocampus histology was comparable with that of the control group, in which a large number of blue stained neurons and a small number of positive apoptotic cells were found. The hippocampal neurons of the rats appeared disordered and loose in group III, and few positive apoptotic cells were found. It was found that the structure of the neurons was disordered, with many positive cells stained brown. The hippocampal neurons were wrinkled and quite loose in group V, and a great quantity of apoptotic neurons were found, as indicated in the Figure by arrows. According to the count, the number of apoptotic neurons in group II was not significantly different compared with the control group. A two-group comparison test showed that the amount of apoptotic neurons in groups III to V was higher than that of group II; group V was the highest (*P* < 0.05).

### Correlation coefficient matrix of plasma lipids and cerebral parameters

**Fig. 4:**
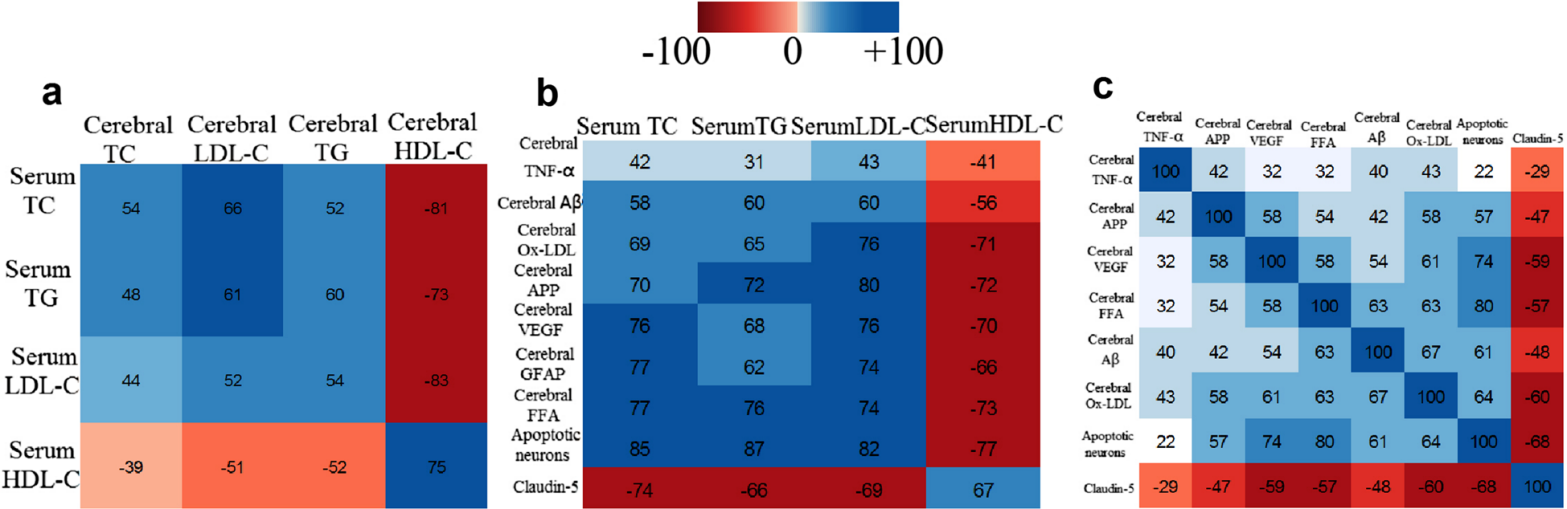
Correlation coefficient matrix of plasma lipids and cerebral parameters. **a** Correlation coefficient matrix of plasma lipids and cerebral lipids. **b** Correlation coefficient matrix of plasma lipids and cerebral parameters. **c** Correlation coefficient matrix between cerebral parameters. The dark areas indicate a strong correlation, and the lighter colored regions are relatively weak

We conducted a correlation analysis of plasma lipids and cerebral parameters and represent the outcome in a matrix diagram [13] (Fig. 4). This matrix shows that the serum TC, TG and LDL-C positively correlated with cerebral TC, TG and LDL-C and negatively correlated with cerebral HDL-C in rats (*P* < 0.01). In addition, serum TC, TG and LDL-C all positively correlated with cerebral TNF-α, Aβ, ox-LDL, APP, VEGF, GFAP, FFA and the number of apoptotic neurons, but negatively correlated with the expression of tight junction protein Claudin-5 (*P* < 0.05). The levels of TNF-α, APP, VEGF, FFA, Aβ, ox-LDL and the number of apoptotic neurons in the brains positively correlated with each other, and they negatively correlated with Claudin-5 (*P* < 0.05).

## Discussion

It is widely accepted that hyperlipidemia may lead to atherosclerosis, and hyperlipidemia has been identified as an independent risk factor for coronary heart disease and ischemic stroke [14]. Extensive epidemiologic and experimental studies have supported the conception that hyperlipidemia may cause nervous system-related diseases [15, 16]. We evaluated the hyperlipidemia model with reference to the Deepa modeling method (normal rat chow supplemented with 4% cholesterol, 1% cholic acid and 0.5% thiouracil, also called the CCT diet) [9]. In rats that were fed the CCT diet for 1 week, the levels of serum TC, TG and LDL-C increased by 2.40, 1.29 and 1.75 times,(*P* < 0.05), respectively, and the levels of serum HDL-C decreased by 0.26 times (*P* < 0.05). Factorial analysis shows that the severity of hyperlipidemia was aggravated with the extension of the CCT diet.

Brain tissue is composed of neurons and glial cells, and this tissue is a significant integration center for body movements, sensations and contact functions. Immunologic cells, such as microglials and astrocytes (Ast), secrete a variety of cytokines (TNF-α, IL-1, IL-6) as immune responses when pathological damage occurs [17]. Astrocytes play a crucial role in maintaining the physiological functions of the blood brain barrier and regulating the metabolism of lipids in the brain [18]. GFAP is a kind of specific reactive monomer protein of astrocytes, and the expression of GFAP reflects the activity of the astrocytes [19]. Our study showed that the levels of cerebral TG, IL-1 and TNF-α, as well as the expression of GFAP, increased in rats after being fed the CCT diet for 1 week. Cerebral TG was positively correlated with serums TC and TG (*P* < 0.05) according to the correlation analysis, which suggests that the cerebral TG was associated with the increase in serums TC and TG. Also, there was a positive correlation between the expression of GFAP and the concentration of cerebral TG, IL-1 and TNF-α (*P* < 0.05). We therefore propose that brain TG may stimulate the proliferation of astrocytes and promote the secretion of inflammatory cytokines TNF-α and IL-1, causing the destruction of brain tissue. We found that in rats that had consumed the CCT diet for 1 week, the expression of tight junction protein Claudin-5 and the number of neurons showed no difference as compared with the control group. Therefore, we concluded that the permeability of the blood brain barrier did not change, and the neurons were not injured, during the first week of the CCT diet.

FFA in the serum reflects the level of lipid metabolism and mediates cell damage caused by oxidative stress [20–24]. Brain FFA content is extremely low under normal conditions, but it increases when circulation disorders occur in the brain [25, 26]. The results of our experiment in the model with the CCT diet rats show that the level of cerebral FFA, the expression of APP and the number of apoptotic neurons improved in the second week (*P* < 0.05). Cerebral FFA had a positive correlation with serum TG and the number of apoptotic neurons (*P* < 0.05) according to our correlation analysis, which indicated that an increase in brain TG could lead to the increase of FFA levels in the brain, which would result in damage to the neurons.

Ox-LDL plays a crucial role in inducing atherosclerosis [27, 28]. It is formed by the oxidation of LDL, and extensive studies indicate that it can damage the cytoskeleton, stimulate the apoptosis of cells and damage the endothelial cells through a variety of signaling pathways [29–31]. This oxidation reaction promotes the precipitation of Aβ characterized by Alzheimer's senile plaques [32], and it triggers amyloid lesions in the vascular endothelium. Studies have suggested that Aβ may be induced by inflammation and oxidative stress to express the oligomer with neurotoxicity [33–35].

VEGF is a kind of glycoprotein closely related to cerebral vascular disease, and it has a significant ability to promote vascular endothelial cell division and proliferation, promote the growth of plaque, stimulate the inflammation response and change the permeability of the blood brain barrier [36, 37]. Claudin-5 is a tight junction protein that participates in the formation of the blood brain barrier [38], and the permeability of the blood brain barrier would be damaged if the expression of Claudin-5 were reduced. This would destroy the stability of the internal environment in the central nervous system, which may ultimately injure the brain tissue [39].

In rats that had consumed the CCT diet for three weeks, the levels of ox-LDL, Aβ and VEGF increased in the brains (*P* < 0.05) while the expression of tight junction protein Claudin-5 and the count of hippocampal nerve cells decreased (*P* < 0.05). The related correlation analysis shows the positive correlations among the following factors: cerebral ox-LDL and FFA (*P* < 0.05); cerebral TC and Aβ, VEGF (*P* < 0.05); the amount of apoptotic neurons and cerebral ox-LDL, Aβ (*P* < 0.05). We also found there was a negative correlation between cerebral VEGF and Claudin-5 (*P* < 0.05). This implies that FFA may induce the production of ox-LDL, while TC stimulates the precipitation of Aβ and the expression of VEGF in the brain. Ox-LDL, Aβ and VEGF in the brain may destroy the permeability of the blood–brain barrier by damaging the vascular endothelial cells, aggravating the injury and apoptosis of neurons as a result.

In the present study, we found that the levels of cerebral TC and LDL-C in rats reached the highest value in the rats that had consumed the CCT diet for four weeks (*P* < 0.05). In this group, the level of cerebral IL-1, TNF-α, FFA, ox-LDL, Aβ, VEGF and the expression of protein GFAP and number of apoptosis neurons were maintained at a higher level (*P* < 0.05), while the expression of protein Claudin-5 remained at the lowest level (*P* < 0.05). Correlation analysis indicates a positive correlation in the rat brains between the levels of cerebral TC, LDL-C and the expression of GFAP (*P* < 0.05), as well as with serum TC (*P* < 0.05). We surmise therefore that the large number of astrocytes that proliferated released an excess of inflammatory cytokines (IL-1, TNF-α), resulting in damage to the cerebral vascular endothelial cells and neurons; this result increased in correlation with the duration of hyperlipidemia in the rats. On the other hand, the levels of brain Aβ and VEGF increased continuously under the stimulation of abnormal brain fats, causing injury to the cerebrovascular tissue and changing the permeability of the blood brain barrier. We thus infer that serum TC and LDL-C may enter the brain through the impaired blood brain barrier and stimulate the astrocytes, which in turn damages the cerebral vessels and neurons.

Because of the lethality of removing the brain of rats, the repeated measures design cannot be used to analyze the duration time of hyperlipidemia on cerebral lipids, vessels and neurons in rats, which may represent a potential limit of our study. We adopted the two-way ANOVA factorial design to deal with the problem, and analyzed the factors of duration and the type of diet on rats significantly.

## Conclusion

In summary, it was found that hyperlipidemia significantly aggravated the level of TG in the brains of rats fed a CCT diet, which stimulated astrocytes to release inflammatory cytokines TNF-α and IL-1, which had direct toxic effects on the cerebral vessels and neurons. The elevated TG levels produced a large number of FFA in the process of its metabolism, and this aggravated the injury to cerebral vessels and neurons by enhancing the oxidation reaction and promoting the production of ox-LDL modified by LDL. We believe that the damage caused by hyperlipidemia to the cerebral vessels and neurons was aggravated with the extension of time the rats consumed the CCT diet. We associate these results with the precipitation of Aβ, the expression of VEGF, the reduction of tight junction protein Claundin-5 and changes in the permeability of the blood brain barrier, all of which were induced by the gradual elevation of levels of TC and LDL-C in the rat brains.

## Abbreviations

APP: A-beta precursor proteins
Aβ: Amyloid beta
ELISA: Enzyme-linked immunosorbent assay
FFA: Free fatty acids
GFAP: Glial fibrillary acidic protein
HDL-C: High density lipoprotein cholesterol
IL-1: Interleukin-1
IL-6: Interleukin-6
LDL-C: Low density lipoprotein cholesterol
ox-LDL: Oxidized low density lipoprotein
TC: Total cholesterol
TG: Total triglycerides
TNF-α: Tumor necrosis factor alpha
TUNEL: Terminal deoxynucleotidyl transferase-mediated dUTP-biotin nick end labeling assay
VEGF: Vascular endothelial growth factor
